# High-Intensity Interval Circuit Training Versus Moderate-Intensity Continuous Training on Functional Ability and Body Mass Index in Middle-Aged and Older Women: A Randomized Controlled Trial

**DOI:** 10.3390/ijerph16214205

**Published:** 2019-10-30

**Authors:** Ismael Ballesta-García, Ignacio Martínez-González-Moro, Jacobo Á. Rubio-Arias, María Carrasco-Poyatos

**Affiliations:** 1 Physical Exercise and Human Performance Research Group, Universidad de Murcia, 30002 Murcia, Spain; Ismael.b.g@um.es (I.B.-G.); igmartgm@um.es (I.M.-G.-M.); 2 Department of Physiotherapy, Universidad de Murcia, 30002 Murcia, Spain; 3 Department of Physical Activity and Sport Sciences, UCAM Research Centre for High Performance Sport. Department of Health and Human Performance, Universidad Politécnica de Madrid (UPM), 28040 Madrid, Spain; jararias@ucam.edu; 4 Department of Education, Health and Public Administration Research Center, Universidad de Almería, 04120 Almería, Spain

**Keywords:** high-intensity interval training, circuit training, older, middle-aged, women, functional ability

## Abstract

The literature suggests that high-intensity interval training (HIIT) is more effective than moderate-intensity continuous training (MICT) to improve functional ability. However, there is no evidence on including HIIT in a circuit programme (HIICT). Our objective was to determine what type of training (HIICT or MICT) induces greater adaptations in the functional ability and body mass index of middle-aged and older women. The study used a quasi-experimental randomized controlled trial with 54 participants (age = 67.8 ± 6.2 years). Participants were randomly allocated to HIICT (*n* = 18), MICT (*n* = 18) or a non-exercise control group (CG; *n* = 18). The participants in the HIICT or MICT groups trained twice a week (1 h/session) for 18 weeks. Forty-one subjects were analysed (HIICT; *n* = 17, MICT; *n* = 12, CG; *n* = 12). Five subjects presented adverse events during the study. Strength, gait, cardiorespiratory fitness, balance and body mass index were measured. A significant training x group interaction was found in the arm curl test, where HIICT was statistically better than MICT and CG. Likewise, HIICT was statistically better than the CG in the BMI interaction. In lower limb strength, gait/dynamic balance and cardiorespiratory fitness, both HIICT and MICT were statistically better than the CG. In conclusion, HIICT generated better adaptations in upper limb strength than MICT. Likewise, HIICT generated better adaptations in body mass index than CG. Finally, both HIICT and MICT had a similar influence on strength, cardiorespiratory fitness and gait/dynamic balance.

## 1. Introduction

The world population aged over 65 years has increased considerably in recent years [1]. Due to hormonal changes (i.e., menopause), the effect of ageing in women is resulting in chronic diseases, functional dependence, the prevalence of sarcopenia and frailty and the risk of falls and injuries, increasing the risk of hospitalization and mortality [2,3,4,5,6]. This generates significant economic cost for health systems to prevent or treat age-related chronic diseases and dependency situations [1].

Recently, physical exercise has become relevant for the maintenance of health and functional ability in middle-aged and older women [4,7,8], functional ability being understood as the capacity to carry out activities of daily living [9]. This has been evidenced in recent studies indicating that maintaining high levels of strength, balance, gait and cardiorespiratory fitness are the main objectives for the optimal achievement of the basic and instrumental daily living activities for middle-aged and older women, as well as to delay the onset of sarcopenia and frailty [3,4,7,10]. Similarly, physical exercise also helps maintain healthy body mass index (BMI) values, which is an important factor in their relationship to chronic diseases that cause dependence in older women populations [11].

For these reasons, governments and the healthcare system are implementing physical exercise programmes for middle-aged and older people at day centres [12]. As the training sessions are usually in groups, circuit training is one of the most common types of training [13,14,15]. Circuit training consists of performing a series of exercises for all muscle groups, allowing strength and cardiorespiratory fitness to be influenced simultaneously [16,17]. Body weight-based circuit training can be considered a functional training method because they are based on the imitation of daily living activities in which only body weight is used (i.e., body weight squats and lunges).

Some research involving the elderly has shown that moderate-intensity circuit training (MICT) has positive effects on strength, cardiorespiratory fitness, balance, body composition and quality of life [18,19,20,21,22]. Furthermore, during recent years a new type of training called high-intensity interval training (HIIT) has been included as an alternative method of training in healthy people and as part of the treatment of pathologies, especially in cardiac rehabilitation programmes [23,24,25]. HIIT consists of intermittent short high-intensity work periods (85–100% of maximal oxygen uptake) and relative rest periods [26]. Some research has shown strong evidence that HIIT is an effective method for improving maximal oxygen uptake [23], strength [27,28], cardiorespiratory fitness [27,29], gait [27,30] and body composition [31] in older people with pathologies. In fact, there is a trend that indicates that health indices and markers are more favourable after HIIT than after MICT [26]. Otherwise, this is still not clear regarding other functional parameters [32,33,34].

However, although the benefits of HIIT are known, there is little evidence on including HIIT methodology in circuit training (HIICT) in healthy middle-aged and older women. In this sense, HIICT is an alternative form of training for improving physical condition and performance in daily living activities due to integrated multiplanar-based movements (squatting, pulling, etc.) performed at maximal speed, which makes it very functional and with high transfer in older adults [14,35].

For these reasons, the main objective of the present study was to determine what type of training (HIICT or MICT) induces greater adaptations in the functional ability and BMI of middle-aged and older women. Based on the previous research, our hypothesis was that HIICT would significantly improve the strength, gait, cardiorespiratory fitness, static and dynamic balance and BMI in middle-aged and older women. In addition, it was hypothesized that HIICT would induce greater adaptations in these variables than MICT.

## 2. Materials and Methods 

### 2.1. Design 

This was an 18-week quasi-experimental randomized controlled trial in which independent older women were assigned to an HIICT group (*n* = 18), an MICT group (*n* = 18) or a no-exercise control group (CG; *n* = 18). The trial design followed CONSORT guidelines and was approved by the University of Almería Bioethics Committee (UALBIO2019/006). The study was registered prospectively with ClinicalTrials.gov (NCT03840330).

### 2.2. Participants 

A total of 90 older women (67.8 ± 6.2 years) were invited to participate in the study. Recruitment was from September to December of 2017 from elderly day care centres in Murcia (Spain). A general medical evaluation was performed to ensure the women were physically and mentally able to participate in the exercise programmes. The inclusion criteria were: 55–85-year-old women who were physically able to develop the activities of daily living according to the scales of Lawton and Brody [36] and Katz [37], with no positive answers in the physical activity readiness questionnaire or with a positive answer in Item 6, in order to include women with controlled hypertension and without cardiac, respiratory or joint diseases that could interfere in carrying out the exercise programmes. The exclusion criteria were: Women who were currently participating or had previously participated in a similar exercise programme in the past three months and women with uncontrolled hypertension. The participants also had to maintain at least 80% compliance with the exercise session. All participants signed a consent form before the beginning of the study and the data were collected at the daycare centres.

### 2.3. Interventions 

The participants allocated to HIICT or MICT were required to train twice a week (1 h per session) for 18 weeks from January to May 2018. The women assigned to CG were encouraged to maintain their normal physical activity habits. The HIICT and MICT exercise programmes were conducted by the same accredited exercise expert who was certified in therapeutic physical activity.

The programmes were divided into a 2-week familiarization period and four 4-week mesocycles that were designed to be progressively more challenging. The sessions were given in three phases: (1) the warm-up, (2) the HIICT or MICT exercise programmes and (3) the cool-down. Both the MICT and HIICT were focused on the same movements of the lower limbs, combined with the movements of the upper limbs with or without external load. However, MICT involved moderate speeds, with the objective of working at 9–14 points of rating of perceived exertion (RPE) and HIICT involved high speeds with the aim of working at 14–18 point of RPE. An example of the training and exercise progression implemented is given in Table 1 and Table 2, respectively.

Intensity was controlled using the Börg scale of perceived exertion. This scale establishes a numerical code ranging from 6 to 20 points, where 6 is “very, very light” and 20 is “maximal exertion”, to determine the level of effort and intensity of the exercise during a training session [7]. The first 2 weeks of the study were used to familiarize the participants with the scale. During these familiarization sessions, heart rate monitors were used to associate fatigue with the RPE values. From the first mesocycle, in order to control that the participant worked at the established intensity, they were asked what their RPE were. This question was asked after each block of work and rest in HIICT and every 5 min in MICT. In the MICT group, the sessions began at a moderate intensity (6 or 7 points) and finished at a moderate-to-vigorous intensity (12–14 points). In the HIICT group, 6–12 intervals of moderate-to-vigorous intensity (12–14 points) and high intensity (16–18 points) were rotated in each session. In addition, the duration of the work intervals was 1–1.5 min and the duration of the rest intervals was 2–2.5 min.

### 2.4. Outcomes 

Functional ability, measured with the senior fitness test [38], was the primary outcomes of this study. Secondary outcomes were body mass index and handgrip strength. Testing was performed in all participants before and after the exercise intervention programmes. Pre-tests in January 2018 and post-tests in May 2018, were accomplished over a 1-week period.

Upper limb strength was determined by the 30-second arm curl test (ACT). The participants performed the greatest number of full flexions to extension elbow with a 2-kg dumbbell in 30 s. The test was realized twice with each arm [38].

The lower limb strength was determined by the 30-second sit-to-stand (STS-30). The participants were seated, feet resting on the floor and hands on the opposite shoulder. They were asked to stand up and sit down as many times as possible in 30 s. This test was only performed once [38].

The timed up and go test (TUG) was administered to evaluate the gait. This requires the participant to get up from a chair without armrests, walk 3 m, turn around a cone, walk back to the chair and sit back down. Timing started upon the instructor’s ”go” and stopped when the participant returned to the initial position. The test was executed twice, in addition to an untimed trial. The best time was recorded [38].

The 6-minute walk test (6MWT) was performed to evaluate the cardiorespiratory fitness. The patients were asked to walk as far as possible in a 42-metre indoor corridor. They were allowed to stop during the test if necessary. The distance walked and the heart rate at the beginning and at the end of the test were recorded. The pre–post difference in heart rate during the 6MWT was registered [38].

Balance was assessed using the one-leg standing test (OLS). This test measures the time a participant is able to stand on one leg without support. The time was stopped when 30 s had elapsed or when the standing foot shifted or the lifted foot was placed on the ground. The OLS was realized twice with each leg. The best value for each one was considered in the analysis [38].

The maximal handgrip strength was measured on a Takei dynamometer (TKK 5001). The participants were standing with the arm fully stretched and the wrist in neutral position. The maximal value from three trials from both hand was recorded [39].

Height and weight were measured using an electronic balance and a height rod (Seca 768), respectively, and body mass index (BMI) was calculated according to the formula: BMI = kg/m^2^.

### 2.5. Sample Size and Power

The calculations to establish the sample size were performed using Rstudio 3.15.0 software (Boston, United States). The significance level was set at *p* ≤ 0.05. According to the mean standard deviation established for the 6MWT in a previous study [40] (SD = 53.3 m) and an estimated error (*d*) of 14.2 m, a valid sample size for a 95% confidence interval (CI) was 54 (*n* = *CI*^2^ × *d*^2^/*SD*^2^). A total of 54 women completed the trial. The final sample size for each group obtained in our study (HIICT = 17, MICT = 12, CG = 12) will provide powers of 81%, 65% and 65%, respectively, if between and within a variance of 1.

### 2.6. Randomization and Blinding

A block randomization method was chosen to allocate participants to the groups in equal sample sizes (HIICT, MICT and CG, *n* = 18). The block size was determined according to the statistical power provided. The blocks were chosen randomly to determine the participants’ assignment into the groups. Following Kim [41], a randomization sequence was created using Excel 2016 (Microsoft, Redmond, WA, USA), with a 1:1 allocation via a random number table. Owing to the difficulty of blinding participants and instructors in the exercise trials, only the research staffs performing the assessment and statistical analysis were blinded to group assignment. The chosen allocation concealment method selected was central allocation.

### 2.7. Statistical methods

The statistical analyses were conducted using Jamovi (Jamovi Project 2018, version 0.9.1.7; https://www.jamovi.org) and Rstudio 3.15.0 software (Boston, United States). Prior to the data analysis, the Kolmogorov-Smirnov test was used to determine the normal distribution of the variables. Levene’s test was also performed to determine the homogeneity of variance. The descriptive data are presented as the mean ± SD and range. The intention-to-treat analysis using the last observation for missing data was conducted. To compare variables before the intervention, the analysis of variance (ANOVA) for repeated measures was calculated (general linear model). To compare variables after the intervention, the analysis of covariance (ANCOVA) was used, with baseline values included as co-variables in order to adjust for potential baseline differences in the dependent variables. The age was also included as a co-variable because of the wide range considered in the present study (55–85 years). The standardized mean differences (Cohen’s effect size) were calculated together with the 95% confidence intervals [39]. An effect size (ES) value of 0.20 indicates a small effect, 0.60 a moderate effect, 1.2 a large effect, 2.0 a very large effect and 4.0 a near-perfect effect [42]. The level of significance was set to *p* ≤ 0.05.

## 3. Results

**Figure 1:**
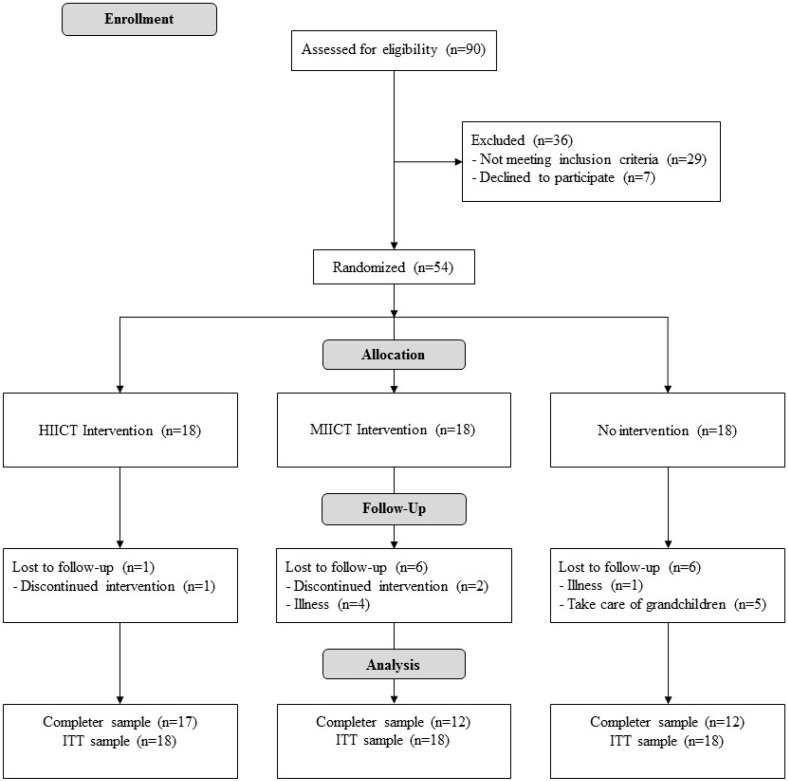
Flow diagram of the progress of the randomized trial.

Figure 1 illustrates the participant flow during the protocol. Twenty-nine women did not meet the inclusion criteria and seven declined to participate. In total, 54 women were actually enrolled in the study and randomly distributed into HIICT, MICT and CG. Finally, 41 participants (HICT, *n* = 17; MICT, *n* = 12; GC, *n* = 12) completed the study. The participants completed 84% and 87% of the training in the HIICT and MICT groups, respectively. The trial ended in May 2018. The baseline characteristics of the participants in the three groups are shown in Table 3.

### 3.1. Inter-group results

The inter-group results in the primary and secondary outcomes are presented in Table 4 and Table 5. The main analysis of these outcomes indicates that there was a significant training x group interaction (*p* < 0.001) in the ACT, STS-30, TUG, 6MWT and BMI.

- In the ACT, HIICT was statistically better than MICT (dif = 6.6 rep, *t* = −3.905) and CG (dif = 9.3 rep, *t* = −2.986).
- In the 30-STS, HIICT was statistically better than CG (dif = 5.6 rep, t = −5.84) and MICT was also statistically better than CG (dif = 3.2 rep, *t* = −3.87).
- In the TUG, HIICT was statistically better than CG (dif = −0.78 sec, *t* = 4.03) and MICT was also statistically better than CG (dif = −0.23 sec, *t* = 4.244).
- In the 6MWT, HIICT was statistically better than CG (dif = 43 m, *t* = −3.631) and MICT was also statistically better than CG (dif = 55 m, *t* = −3.719).
- Regarding the BMI, HIICT was statistically better than CG (dif = −1.4 kg/m^2^, *t* = 3.58).

### 3.2. Intra-Group Results

The additional intra-group analysis (Table 6 and Table 7) shows a significant improvement (*p* < 0.001) in the STS-30, TUG and 6MWT for both HIICT and MICT. Regarding the ACT, either HIICT or CG showed significant improvements (*p* = 0.022 and *p* < 0.001, respectively). On the other hand, a significant decrease was obtained in the STS-30 and TUG for CG (*p* < 0.001 and *p* = 0.016, respectively), and in the right OLS (*p* = 0.024) for HIICT. With regard to the BMI, HIICT showed a significant reduction (*p* = 0.035), whereas CG showed a significant increase (*p* = 0.019).

Regarding safety, there were registered adverse events in MICT and CG groups. Four women in the MICT group and one in CG were lost to follow-up due to eye surgery, foot surgery, clavicle fracture and two hip fractures after a fall. These adverse events did not occur during the exercise classes.

## 4. Discussion

The present study investigated what type of training (HIICT or MICT) induces greater adaptations in strength, gait, cardiorespiratory fitness, static balance and BMI of middle-aged and older women. The main finding of this study was that HIICT was more effective than MICT for improving upper limb strength in healthy middle-aged and older women. In addition, both HIICT and MICT were also effective for improving lower limb strength, gait and cardiorespiratory fitness. The secondary analyses suggest that HIICT is an effective training method to reduce BMI after 18 weeks of training. To the best of the authors’ knowledge, this is the first randomized controlled trial that has evaluated the effects of HIICT on the functional ability in healthy middle-aged and older women.

In relation to muscle strength, the present study shows a significant improvement in the ACT and STS-30 after the 18-week training period in both the HIICT and MICT groups, with significant differences between them and CG. There were also significant differences between HIICT and MICT in the ACT. On the other hand, although HIICT and MICT were not significantly different in the STS-30, a large effect size in HIICT (ES = 1.98) and a moderate effect size in MICT (ES = 1.05) were obtained. In line with our results, Boukabous et al. [43] obtained significant differences in the ACT between HIIT and MICT after 8 weeks of training in older women. In the same way, García-Pinillos et al. [44] and Adamson et al. [45] obtained significant improvements in lower limb strength after 12 weeks of concurrent high-intensity interval and endurance training and a high-intensity interval cycling programme, respectively, in samples composed by men and women. However, these last two studies did not compare the groups with different training intensity.

The upper limb strength results in this study could be associated with the training methodology conducted in HIICT. Although HIICT and MICT contained similar movements in the exercises, execution speed was superior in HIICT, which could lead to neural adaptations (i.e., a greater recruitment of motor units and ST muscle fibres and a decrease in the co-activation) and consequently, a better gain in muscle strength [26]. This is in the line with Correa et al. [46], who compared three types of training in older women, concluding that rapid strength training could be more effective than other types of training for the rapid-force development of muscle. Likewise, it is important to emphasize the importance of the results because the strength of the upper body has been related to the quality of life for women over 60 years of age [47].

On the other hand, it is not clear why there were no significant differences in lower limb strength between HIICT and MICT. The lack of differences could be influenced by the standing position during the sessions in both groups. Likewise, with the duality of the movement and the complexity of the tasks, it is possible that a higher speed during HIICT was not achieved in this case. However, even though MICT improved the strength of the lower limbs, in order to gain strength benefits in the upper and lower limbs from both training methods, this study suggests that HIICT may be used as a time-efficient intervention to increase the strength of both upper and lower limbs.

Regarding the analysis results for gait and dynamic balance, the present study showed a significant improvement in the TUG for both HIICT and MICT groups, with significant differences between them and CG. Although there has not been any research comparing these training methods in older women, these findings are in accordance with several other studies relating both HIIT [45,48,49] and MICT [50,51,52] interventions with improvements on gait. For example, García-Pinillos et al. [44] noted a decreased of 9% in the time to walk 10 m after a 12-week concurrent high-intensity interval strength and endurance training programme in healthy older people. In contrast to our results, a study oriented to suspension training obtained significant differences in the performance of the TUG in HIIT compared to MICT [49]. One possible reason for our results may be precisely the increase in lower limb strength and the nervous system adaptations [53]. The execution of exercise when standing could contribute to assist neural adaptation and thus generate a positive transfer to dynamic balance, gait and lower limb strength [48,54]. The improvement shown in this test has special relevance because it is related to avoiding falls in healthy elderly people. The literature reflects that postural balance during gait is one of the most relevant variables in relation to falls [55]. Similarly, gait speed seems to be a predictor of autonomy in the activities of daily living [56].

For cardiorespiratory fitness, significant improvements were obtained on the 6MWT for both HIICT and MICT, with a moderate effect size in HIICT (ES = 0.84) versus a small effect size in MICT (ES = 0.55). Furthermore, this result is powered by a small effect size in the mean heart rates on HIICT (ES = 0.32) versus a trivial effect size in MICT (ES = 0.13). In line with our results, Boukabous et al. [43] showed that both types of training provided similar improvements in the performance of the 6MWT, even though the HIIT protocol was designed to represent half the duration of MICT (and energy expenditure). On the other hand, Jaureguizar et al. [57] compared the performance on 6MWT after HIIT or MICT, obtaining significant differences in favour of HIIT. The relevance of this improvement is due to the relation of cardiorespiratory fitness with the autonomy to perform daily life activities and prevent or delay frailty in older people [58]. In addition, Fraga et al. [59] showed positive influences of cardiorespiratory fitness on the quality of life of the elderly after a 16 weeks of aerobic training.

Finally, a recent meta-analysis by de Nardi et al. [60] evaluated BMI in a total of 120 prediabetic participants without significant differences between HIIT and MICT. Contrary to our results, the meta-analysis refers to a decrease in BMI after the HIIT and MICT programmes. In the same way, a study with cycling did not find a significant decrease in BMI after HIIT in postmenopausal women with diabetes [61]. Similarly, Ramos et al. [62], in a recent study involving overweight participants with and without diabetes, described no significant improvements in BMI after 16 weeks of two different HIIT interventions, without any significant difference between these and MICT.

The strength of this study was the positive effects of HIICT on the functional ability and BMI of healthy middle-aged and older women. The clinical implications of the present study are related to the importance that HIICT could have as an effective method to improve the functional ability and BMI of middle-aged and older women and, consequently, the autonomy to carry out daily living activities and an improved quality of life. Moreover, the feasibility of this kind of circuit training provides the possibility to be easily implemented and with a low-cost material.

Although this study has demonstrated that 18 weeks of HIICT can produce the improvements in strength, gait speed and cardiorespiratory fitness, as well as BMI, it is not without its limitations. Firstly, the non-blinding of participants and instructors and the broad age range of the sample should be considered limitations of this study. Secondly, this trial involved a small number of participants and a larger sample size would have helped to quantify more accurately the changes using this exercise training. Finally, the Börg scale was used to assess exercise intensity. Although the heart rate is more accurate, it was only used during the familiarization phase because the Börg scale is a more useful and practical tool to guide exercise intensity in daily practice.

## 5. Conclusions

The main results obtained in the present research indicate that both HIICT and MICT similarly influenced the strength, cardiorespiratory fitness and gait/dynamic balance variables analysed, except for: (1) the upper limb strength, where the HIICT generated better adaptations than MICT; (2) the BMI, where the HIICT generated better adaptations than CG. These results contribute to improved autonomy in the development of daily living activities as well as to prevent the risk of sarcopenia, frailty and cardiovascular diseases in older women.

## Figures and Tables

**Table 1:** Training progression for HIICT and MICT group.

Mesocycle	Group	Volume (min)	Work Effect (min)	Intensity (p)	Density (w/r)
Mesocycle 1 (weeks 3–6)	HIICT	18–32 (6–8 intervals)	6–12	W: 14–15 Börg R: 7–8 Börg	0.5–1
	MICT	18–32	18–32	9–12 Börg	1
Mesocycle 2 (weeks 7–10)	HIICT	32–40 (9–10 intervals)	12–15	W: 15–16 Börg R: 7–8 Börg	0.5–0.6
	MICT	32–40	32–42	9–12 Börg	1
Mesocycle 3 (weeks 11–14)	HIICT	40 (8–10 intervals)	10–15	W: 16–18 Börg R: 9–10 Börg	0.6
	MICT	40	40	11–13 Börg	1
Mesocycle 4 (weeks 15–18)	HIICT	28–40 (8–12 intervals)	15–18	W: 16–18 Börg R: 10–11 Börg	0.6–0.75
	MICT	40–50	40–50	12–14 Börg	1

HIICT—High-Intensity Interval Circuit Training; MICT—Moderate-Intensity Circuit Training; Börg—Börg Scale of perceived exertion; p—points in Börg Scale; W/r—work/rest ratio.

**Table 2:** Exercise examples for HIICT and MICT groups in each mesocycle.

Mesocycle	HIICT	MICT
Mesocycle 1 (weeks 3–6)	work: grapevine; squat; step-touch; side step; swinging	grapevine; squat; gait; step-touch; side step; mambo cha-cha-cha; swinging
	rest: gait; mambo cha-cha-cha	
Mesocycle 2 (weeks 7–10)	work: grapevine with shoulder and elbow flexion; step-touch with shoulder and elbow flexion; knee-up; chasse; squats with shoulder-up	grapevine with shoulder and elbow flexion; step-touch with shoulder and elbow flexion; gait; knee-up; chasse; squats with shoulder-up; jogging
	rest: gait; jogging	
Mesocycle 3 (weeks 11–14)	work: grapevine with shoulder and elbow flexion and external load; jumping jack; step-touch with shoulder and elbow flexion and external load; chasse with shoulder-up.	grapevine with shoulder and elbow flexion and external load; chasse; jogging; step-touch with shoulder and elbow flexion and external load; chasse with shoulder-up; walking
	rest: jogging; fast walking	
Mesocycle 4 (weeks 15–18)	work: jumping jack with external load; grapevine with shoulder and elbow flexion and external load; knee-up with shoulder and elbow flexion and external load; hamstring curl with shoulder and elbow flexion and external load	step-touch with shoulder and elbow flexion and external load; grapevine with shoulder and elbow flexion and external load; knee-up with shoulder and elbow flexion and external load; hamstring curl with shoulder and elbow flexion and external load; walking; hops
	rest: fast walking; hops	

HIICT—High-Intensity Interval Circuit Training; MICT—Moderate-Intensity Circuit Training.

**Table 3:** Characteristics at baseline (*n* = 56).

Group	*n*	Mean	SD	Min	Max	*p*
Age (years)						
CG	18	67.4	5.71	59	75	0.370
MICT	18	70	8.76	55	86	
HIICT	18	66.3	5.44	57	76	
Body Mass Index (kg/m^2^)						
CG	18	31.2	4.89	20.9	38.4	0.689
MICT	18	30.1	3.08	24.3	35.9	
HIICT	18	30.4	4.13	35.2	37.7	
Arm Curl Test (rep)						
CG	18	20.6	2.96	17.5	28.0	0.102
MICT	18	25.6	5.20	15.5	35.5	
HIICT	18	28.9	5.17	18.5	40.0	
30 Sit-To-Stand (rep)						
CG	18	16.8	2.90	11	22	0.140
MICT	18	13.7	3.38	6	18	
HIICT	18	15.1	2.69	10	19	
Timed Up and Go test (s)						
CG	18	5.89	0.74	4.94	7.46	0.070
MICT	18	6.40	1.23	4.73	9.30	
HIICT	18	6.08	1.31	4.51	9.30	
6-Minute Walking Test (m)						
CG	18	510	59.0	410	590	0.135
MICT	18	502	72.3	355	636	
HIICT	18	564	41.0	455	625	
One Leg Stand test with right leg (s)						
CG	18	19.6	9.09	5.89	30	<0.001
MICT	18	21.6	11.20	3.00	30	
HIICT	18	26.9	7.75	2.00	30	
One Leg Stand test with left leg (s)						
CG	18	15.1	9.10	1.98	30	<0.001
MICT	18	19.9	10.00	2.00	30	
HIICT	18	27.4	6.87	2.00	30	
Right Handgrip Strength (kg)						
CG	18	23.0	4.21	16.0	31.0	0.345
MICT	18	23.6	5.10	16.0	36.0	
HIICT	18	25.8	3.77	20.0	34.5	
Left Handgrip Strength (kg)						
CG	18	21.4	4.06	14.0	29.0	0.904
MICT	18	22.3	4.01	16.5	31.0	
HIICT	18	25.0	3.49	20.0	32.5	
Heart Rate during 6-Minute Walking Test (bpm)						
CG	18	34.9	14.8	15	57	0.684
MICT	18	26.7	14.7	5	59	
HIICT	18	34.8	17.1	−15	65	

CG—Control Group; MICT—Moderate-Intensity Circuit Training; HIICT—High-Intensity Interval Circuit Training. Statistically significant differences at *p* ≤ 0.05 are given in bold.

**Table 4:** ANCOVA adjustments for the primary outcomes on HIICT, MICT and CG.

Group			Increment		ANCOVA Interactions (F, p, ES η²)								
	*n* (ITT)	*n* (Treated)	Mean	SD	Training × Group			Training × Baseline			Training × Age		
					F	*p*	ES η²	F	*p*	ES η²	F	*p*	ES η²
**Arm Curl Test (rep)**													
CG	18	12	1.80	−0.10	8.1	**8.99 × 10^−4 2,3^**	0.192	18.3	**8.51 × 10^−5^**	0.217	9.85 × 10^−3^	0.921	0.000
MICT	18	12	−0.50	−1.14									
HIICT	18	17	2.80	0.28									
30 Sit-To-Stand (rep)													
CG	18	12	−1.90	−0.05	17.42	**1.81 × 10^−6 1,2^**	0.381	6.54	**1.36 × 10^−2^**	0.072	0.353	0.555	0.004
MICT	18	12	3.80	1.47									
HIICT	18	17	5.60	0.47									
Timed Up and Go Test (s)													
CG	18	12	0.36	0.16	11.41	**8.30 × 10^−5 1,2^**	0.281	8.45	**5.42 × 10^−3^**	0.104	0.274	0.603	0.004
MICT	18	12	−0.87	0.05									
HIICT	18	17	−0.78	−0.51									
6-Minute Walking Test (m)													
CG	18	12	−16	−9.50	9.34	**3.57 × 10^−4 1,2^**	0.256	4.42	**4.05 × 10^−2^**	0.060	0.274	0.603	0.004
MICT	18	12	43	0.30									
HIICT	18	17	36	33.9									
One Leg Stand Test with Right Leg (s)													
CG	18	12	1.70	1.91	2.38	0.102	0.086	4.03	**0.050**	0.07	2.48	0.122	0.044
MICT	18	12	1.10	−1.32									
HIICT	18	17	−1.60	1.12									
One Leg Stand Test with Left Leg (s)													
CG	18	12	−3.40	1.80	1.9	0.160	0.069	4.07	**0.049**	0.067	0.017	0.895	0.000
MICT	18	12	0.90	1.00									
HIICT	18	17	−2.60	1.87									

ITT—Intention to treat; SD—Standard deviation; ANCOVA—Covariance analysis; F—estimated variance; CG—Control Group; MICT—Moderate-Intensity Circuit Training; HIICT—High-Intensity Interval Circuit Training. ^1^ Denotes significant difference in MICT compared to CG; ^2^ denotes significant differences in HIICT compared to CG; ^3^ denotes significant differences in HIICT compared to MICT; Statistically significant differences at *p* ≤ 0.05 are given in bold.

**Table 5:** ANCOVA adjustments for the secondary outcomes on HIICT, MICT and CG.

Group	*n* (ITT)	*n* (Treated)	Increment		ANCOVA Interactions (F, *p*, ES η²)								
			Mean	SD	Training × Group			Training × Baseline			Training × Age		
					F	*p*	ES η²	F	*p*	ES η²	F	*p*	ES η²
Body Mass Index (kg/m^2^)													
CG	18	12	0.30	−0.05	6.99	**0.002 ^2^**	0.215	3.02	0.088	0.046	0.217	0.643	0.003
MICT	18	12	−0.10	1.47									
HIICT	18	17	−0.30	0.47									
Right Handgrip Strength (kg)													
CG	18	12	0.60	−0.86	0.881	0.420	0.033	9.72	**3.02 × 10^−3^**	0.155	1.45	0.233	0.028
MICT	18	12	−0.40	0.49									
HIICT	18	17	0.60	−0.68									
Left Handgrip Strength (kg)													
CG	18	12	0.90	0.04	1.46	0.243	0.054	1.39	0.244	0.026	1.13	0.293	0.021
MICT	18	12	−0.20	0.74									
HIICT	18	17	0.60	0.87									
Heart Rate during 6-Minute Walking Test (bpm)													
CG	18	12	−3	−9.50	1	0.375	0.038	13.39	**6.08 × 10^−4^**	0.197	1.74	0.193	0.032
MICT	18	12	2	0.30									
HIICT	18	17	6	33.90									

ITT—Intention to treat; SD—Standard deviation; ANCOVA—Covariance analysis; F—estimated variance; CG—Control Group; MICT—Moderate-Intensity Circuit Training; HIICT—High-Intensity Interval Circuit Training. ^2^ Denotes significant differences in HIICT compared to CG; Statistically significant differences at p ≤ 0.05 are given in bold.

**Table 6:** Intra-group differences for the primary outcomes on HIICT, MICT and CG.

Group	Pre-Training			Post-Training			*p*	95% CI for MD		Cohen’s *d*
	n	Mean	SD	n	Mean	SD		Lower	Upper	
Arm Curl Test (rep)										
CG	18	20.6	2.96	12	22.4	2.86	**2.04 × 10^−4^**	−2.61	−0.99	0.57
MICT	18	25.6	5.20	12	25.1	4.06	0.575	−1.34	2.34	0.09
HIICT	18	28.9	5.17	17	31.7	5.45	**0.022**	−5.09	−0.45	0.52
30 Sit-To-Stand (rep)										
CG	18	16.8	2.90	12	14.9	2.85	**1.40 × 10^−4^**	1.07	2.70	0.61
MICT	18	13.7	3.38	12	17.5	4.85	**3.20 × 10^−4^**	−5.65	−2.01	1.05
HIICT	18	15.1	2.69	17	20.7	3.16	**1.00 × 10^−6^**	−7.13	−4.08	1.98
Timed Up and Go Test (s)										
CG	18	5.89	0.74	12	6.25	0.89	**0.016**	−0.64	−0.07	0.46
MICT	18	6.40	1.23	12	5.53	1.28	**0.001**	0.38	1.34	0.66
HIICT	18	6.08	1.31	17	5.30	0.80	**0.009**	0.21	1.35	0.57
6-Minute Walking Test (m)										
CG	18	510	59.0	12	494	49.5	0.058	−0.60	33.39	0.25
MICT	18	502	72.3	12	545	72.6	**3.09 × 10^−4^**	−61.94	−22.46	0.55
HIICT	18	564	41.0	17	600	74.9	**0.036**	−8.65	−2.57	0.84
One Leg Stand Test with Right Leg (s)										
CG	18	19.6	9.09	12	21.3	11.00	0.233	−4.57	1.19	0.17
MICT	18	21.6	11.20	12	22.7	9.88	0.237	−3.06	0.81	0.99
HIICT	18	26.9	7.75	17	25.3	8.87	**0.024**	0.24	3.10	0.20
One Leg Stand Test with Left Leg (s)										
CG	18	15.1	9.1	12	11.7	10.90	0.129	−1.08	7.79	0.35
MICT	18	19.9	10.0	12	20.80	11.00	0.486	−3.54	1.75	0.08
HIICT	18	27.4	6.87	17	24.80	8.74	0.124	−0.80	6.05	0.36

SD—Standard deviation; CG—Control Group; MICT—Moderate-Intensity Circuit Training; HIICT—High-Intensity Interval Circuit Training; Statistically significant differences at *p* ≤ 0.05 are given in bold.

**Table 7:** Intra-group differences for the secondary outcomes on HIICT, MICT and CG.

Group	Pre-Training			Post-Training			*p*	95% CI for MD		Cohen’s d
	n	Mean	SD	n	Mean	SD		Lower	Upper	
Body Mass Index (kg/m^2^)										
CG	18	31.2	4.89	12	31.5	5.05	**0.019**	−0.52	−0.53	0.06
MICT	18	30.1	3.08	12	30.0	3.15	0.140	−0.02	0.29	0.03
HIICT	18	30.4	4.13	17	30.1	4.24	**0.035**	0.02	0.60	0.07
Right Handgrip Strength (kg)										
CG	18	23.0	4.21	12	23.6	3.35	0.155	−1.54	0.26	0.13
MICT	18	23.6	5.1	12	23.2	5.59	0.650	−1.38	2.16	0.07
HIICT	18	25.8	3.77	17	26.4	3.09	0.397	−1.80	0.75	0.15
Left Handgrip Strength (kg)										
CG	18	21.4	4.06	12	22.3	4.10	0.219	−2.28	0.56	0.21
MICT	18	22.3	4.01	12	22.1	4.75	0.656	−0.91	1.41	0.05
HIICT	18	25	3.49	17	25.6	4.36	0.229	−1.64	0.42	0.16
Heart Rate during 6-Minute Walking Test (bpm)										
CG	18	34.90	14.80	12	31.7	12.30	0.303	−3.179	9.624	0.21
MICT	18	26.70	14.70	12	28.7	12.30	0.565	−9.184	5.184	0.13
HIICT	18	34.80	17.10	17	40.6	14.20	0.137	−13.582	2.027	0.32

SD—Standard deviation; CG—Control Group; MICT—Moderate-Intensity Circuit Training; HIICT—High-Intensity Interval Circuit Training; Statistically significant differences at *p* ≤ 0.05 are given in bold.
